# Does Screen Time Do More Damage in Boys Than Girls?

**DOI:** 10.7759/cureus.72054

**Published:** 2024-10-21

**Authors:** Konstantine Chakhunashvili, Eka Kvirkvelia, Davit G Chakhunashvili

**Affiliations:** 1 Pediatrics, University of Georgia, Tbilisi, GEO; 2 Obstetrics and Gynecology, Caucasus University, Tbilisi, GEO; 3 Pediatrics, Alte University, Tbilisi, GEO

**Keywords:** development, expressive language delay, parental supervision, screentime, speech

## Abstract

Background

Technological progress, particularly accelerated by the recent pandemic, has led to the digitalization of many aspects of daily life. Consequently, children are increasingly exposed to screen time, raising concerns about its potential impact on early development.

Methods

Four separate questionnaires were developed for different age groups (12-18 months, 19-30 months, 31-48 months, and 49-72 months). Data were collected over a two-month period from three major pediatric facilities, and social media platforms were also utilized to reach participants. The total sample size consisted of 5,137 children.

Results

Exposure to active screen time was found to increase the risk of expressive language delay, with a significant correlation identified between early screen time exposure and expressive language delay. In the 12-18 months age group, the OR was 1.52 (p = 0.008, χ²(1) = 7.08, p = 0.008), while in the 19-30 months age group, the OR was 1.79 (p = 0.0002; χ²(1) = 14.30, p < 0.001).

Conclusions

This study provides significant insights into the complex relationship between screen time exposure and expressive language development in young children. Our findings reveal that early screen time exposure is associated with a higher risk of expressive language delay. The data suggest that children aged 12-30 months are especially vulnerable. However, we cannot definitively state that the amount of average daily screen time is significantly changing the outcome. Conversely, we also found that a lack of screen time exposure may be associated with an increased risk of language delay in children aged 31-48 months, highlighting the nuanced role of screen time in child development. Parental supervision is a critical factor; however, our study indicates that higher levels of supervision do not consistently mitigate the risks associated with screen time across all age groups. Further research is warranted to explore the underlying factors contributing to the observed differences in susceptibility to expressive language delay among genders and to examine the long-term impacts of early screen exposure. By understanding these dynamics, we can better support children’s developmental needs in an increasingly screen-saturated environment.

## Introduction

The advancement of technology and the proliferation of diverse digital devices have significantly increased exposure to digital media and screen time. Screen time refers to the duration spent using devices such as computers, televisions, game consoles, and mobile phones [1]. The World Health Organization and the American Academy of Pediatrics (AAP) recommend that children aged two to four years should be limited to no more than one hour of screen time per day, preferably with a parent engaging in educational content alongside the child [2]. For children under two years old, the AAP advises against any screen time exposure [3]. The National Institute for Health and Care Excellence suggests limiting leisure screen time to two hours daily for children of any age [4]. However, these guidelines are often disregarded, and many children exceed the recommended screen time. A 2017 national study in the United States found that approximately 46% of children under the age of two had been exposed to mobile phone screens at least once [5]. Touchscreen devices are frequently used as “electronic babysitters” to soothe or calm crying or irritable babies [6]. The potential consequences of excessive screen time have drawn increased interest over the past decade, sparking debate among physicians and researchers [4]. Pediatric societies have become particularly concerned about the impact of screen time on language acquisition, as early childhood is a critical period for language development, which is largely fostered through interactions with parents. There is growing concern that screen time may reduce the quantity and quality of these interactions, potentially hindering children's language development [2].

## Materials and methods

A correlational research design was selected for this study. Four anonymous questionnaires, tailored to different age groups - 12-18 months, 19-30 months, 31-48 months, and 49-72 months - were developed using Microsoft Forms (Microsoft Corporation, Redmond, WA, USA). Data collection took place over two months, from September 2023 to early November 2023. To facilitate participation, printed QR code stickers linked to the online questionnaires were placed in three clinics: I. Tsitsishvili Children’s Hospital, Mziurimed, and David Abuladze Georgian-Italian Clinic. Pediatricians at these clinics were instructed to enroll their patients in the study. Additionally, social media platforms were utilized to reach a broader audience. The study was approved by the Ethical Committee of the University of Georgia (study code: UGREC-32-23) and is in accordance with the Declaration of Helsinki. Informed consent was obtained from each parent and/or legal guardian. Selection criteria included the following: (a) age between 12 and 72 months; (b) no previously diagnosed developmental disorder, neurological delay, or any condition affecting development.

Questionnaire

Each questionnaire began with an informed consent form and detailed information about the study. Participants were asked about the child’s initials, date of birth, sex, average daily screen time (regardless of device), percentage of supervised screen time, approximate vocabulary size, and time spent with a parent. Each questionnaire was compiled using well-recognized tools and red flags associated with expressive language delay [7-9].

The questionnaire for children aged 12-18 months included questions about the child’s approximate vocabulary, beyond “dada” and “mama,” whether the child addresses a parent (e.g., “dada,” “mama”), and the presence of gesture communication. For children aged 19-30 months, questions focused on whether the child uses at least five different words for commenting, demanding, or naming objects, combines two words, uses gestures other than waving and pointing, plays with other children or in their proximity, and the percentage of speech intelligible to a stranger.

The questionnaire for children aged 31-48 months assessed the child’s vocabulary size, ability to engage in dialogue with at least two exchanges, ability to ask “what,” “when,” and “where” questions, ability to provide their name when asked, play with other children, name actions in pictures, and learn words from stories, poems, or songs. It also asked the percentage of speech intelligible to strangers.

For children aged 49-72 months, the questionnaire inquired whether the child could tell at least two stories (real or imaginary), answer two to three questions about a story that was read or told to them, follow the rules of playing with others, form sentences of four or more words, and engage in a dialogue with three or more exchanges. It also included questions on speech intelligibility to strangers.

Criteria for expressive language delay

Language delay criteria vary by age. For children aged 12-14 months, language delay is indicated if they do not use “mama” or “dada” to call a parent and do not use gesture communication. At 15-17 months, signs include not using one or two words other than “mama” or “dada,” not using gestures, and not pointing to gain attention or assistance. For children aged 18-23 months, a delay may be present if they do not use at least five words or fail to use gestures to share enjoyment. Between 24 and 36 months, indicators include a vocabulary of fewer than 50 words, absence of two-word combinations, and limited gestures beyond waving and pointing. For those aged 37-48 months, not engaging in dialogue with at least two exchanges, not asking “what,” “when,” and “where” questions, inability to describe actions in pictures, and not stating a name when asked are signs of delay. At 49-58 months, signs include not using sentences of four or more words and only repeating words from songs, stories, or movies. Finally, for children aged 59-72 months, a delay may be evident if they do not answer questions about a book or story or fail to engage in a dialogue with three or more exchanges.

Statistics

Data were analyzed using IBM SPSS Statistics for Windows, Version 26.0 (Released 2019; IBM Corp., Armonk, NY, USA). Descriptive statistics, including means, standard deviations, and frequencies, were calculated to summarize the general characteristics of the sample population. Differences between groups (e.g., children with and without expressive language delay) were assessed using independent sample t-tests for continuous variables (e.g., average daily screen time) and chi-squared tests for categorical variables (e.g., gender distribution). To evaluate the association between screen time and expressive language delay, ORs with 95% CIs were calculated through logistic regression models. An OR quantifies the association between exposure and outcome, comparing the odds of an event occurring in the exposed group versus the unexposed group. An OR of 1 indicates no association, >1 suggests a positive association, and <1 indicates a negative association. Separate logistic regression analyses were conducted for each age group to determine the effect of screen time exposure while adjusting for potential confounders like gender and percentage of supervised screen time. Additionally, subgroup analyses were performed to explore gender-specific effects. A stratified analysis by gender was also carried out to determine if the impact of screen time varied between male and female children within each age category. The interactions between screen time exposure and gender were tested using interaction terms in the logistic regression models. For continuous variables like the average daily screen time duration and percentage of the day spent with a parent, we used independent sample t-tests and ANOVA to identify differences across groups. In cases where the assumptions of normality and homogeneity of variances were violated, Welch’s t-test was applied. Statistical significance was set at p < 0.05 for all analyses. Results are presented in terms of ORs, mean differences, and corresponding 95% CIs. Graphical representations, including bar charts, scatter plots, and forest plots, illustrate the relationships between screen time exposure and expressive language delay, as well as any observed gender differences.

## Results

General information

The total sample size was 5,137, comprising 1,678 unique entries collected via the questionnaire for children aged 12-18 months, 1,497 from the questionnaire for children aged 19-30 months, 1,170 from the questionnaire for children aged 31-48 months, and 792 from the questionnaire for children aged 49-72 months. In total, 2,882 entries were for male children, 2,190 for female children, and 65 entries did not specify the child’s sex.

12-18 months

**Figure 1:**
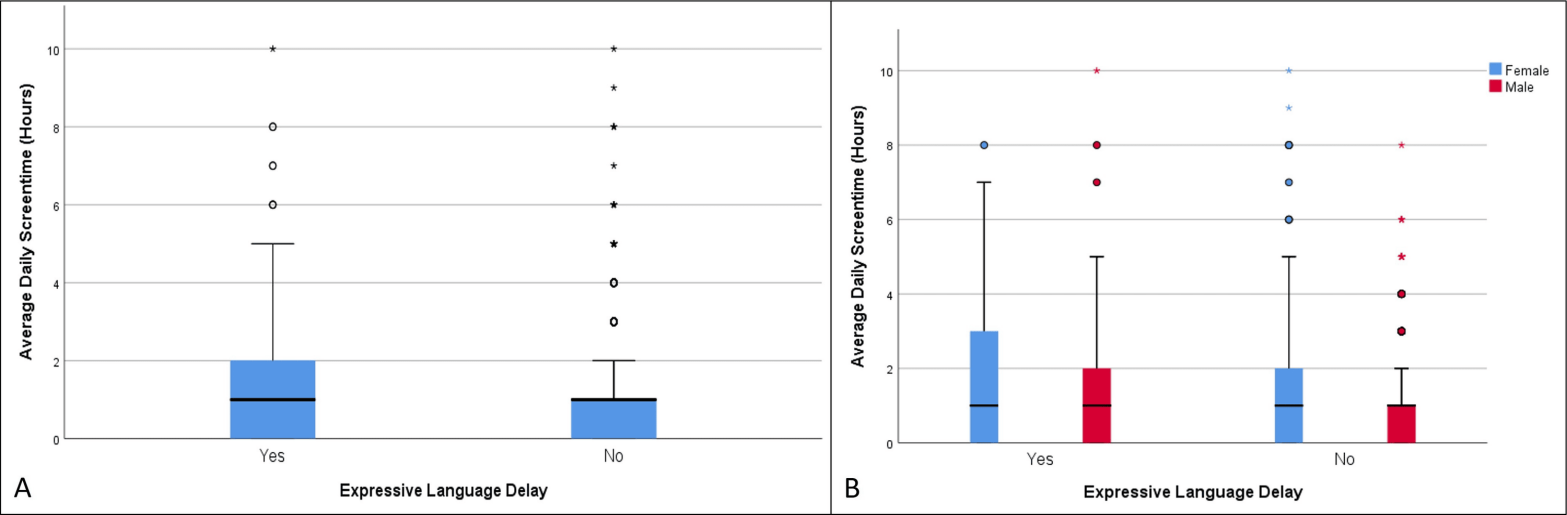
Distribution of average daily screen time in children with and without expressive language delay (A), as well as the same distribution categorized by biological sex (B) (age group 12-18 months)

There were statistically significant differences in the mean values of average daily screen time between children with expressive language delay (1.6 hours) and those without (1.09 hours), t = -4.06, df = 246.18, p < 0.001 (Table 1, Table 2, Figure 1). The OR for developing expressive language delay among those exposed to screen time versus those not exposed was 1.52 (95% CI (1.11, 2.08)), p = 0.008, and a statistically significant correlation was detected between exposure and the development of expressive language delay, χ²(1) = 7.08, p = 0.008. The OR for females was not statistically significant (OR = 1.34, 95% CI (0.80, 2.26)), p = 0.26, whereas for males it was significant (OR = 1.69, 95% CI (1.13, 2.50)), p = 0.0099. Although expressive language delay was observed to be affected by screen time in the combined data, males experienced a greater impact, with a statistically significant difference between males (15.63%) and females (9.44%) resulting in a 6.19% difference (95% CI (2.98, 9.36)), χ²(1) = 14.02, p < 0.0001 (Table 3, Figure 1). It is important to note that, according to the data, girls were exposed to more average daily screen time than boys.

**Table 1:** Average daily screen time (ranging from 0, indicating no exposure, to 10 hours) and the number of children with expressive language delay (age group 12-18 months)

		Expressive language delay		Total
		Yes	No	
Average daily screen time (hours)	0	64	572	636
	1	66	506	572
	2	31	173	204
	3	23	98	121
	4	13	43	56
	5	6	28	34
	6	3	7	10
	7	2	1	3
	8	2	4	6
	9	0	1	1
	10	1	1	2
Total		211	1,434	1,645

**Table 2:** Overview of expressive language delay status, average daily screen time (in hours), percentage of supervised screen time, and percentage of time spent with a parent (age group 12-18 months)

	Expressive language delay	N	Mean	SD	Standard error of the mean
Average daily screen time (hours)	No	1,434	1.09	1.324	0.035
	Yes	211	1.6	1.76	0.121
Supervised screen time by a family member (%)	No	852	63.19	38.751	1.328
	Yes	146	63.25	36.159	2.993
Part of a day spent with a parent (%)	No	1,434	86.25	23.875	0.63
	Yes	212	81.13	27.062	1.859

**Table 3:** Average daily screen time, ranging from 0 (indicating no exposure) to 10 hours, and the corresponding number of males and females with expressive language delay (age group 12-18 months)

Biologic sex			Expressive language delay		Total
			Yes	No	
Female	Average daily screen time (hours)	0	23	267	290
		1	18	247	265
		2	9	82	91
		3	10	46	56
		4	5	28	33
		5	2	11	13
		6	3	3	6
		7	1	1	2
		8	1	3	4
		9	0	1	1
		10	0	1	1
	Total		72	690	762
Male	Average daily screen time (hours)	0	40	303	343
		1	47	256	303
		2	22	91	113
		3	13	52	65
		4	8	15	23
		5	4	17	21
		6	0	4	4
		7	1	0	1
		8	1	1	2
		10	1	0	1
	Total		137	739	876
Total	Average daily screen time (hours)	0	63	570	633
		1	65	503	568
		2	31	173	204
		3	23	98	121
		4	13	43	56
		5	6	28	34
		6	3	7	10
		7	2	1	3
		8	2	4	6
		9	0	1	1
		10	1	1	2
	Total		209	1,429	1,638

**Figure 2:**
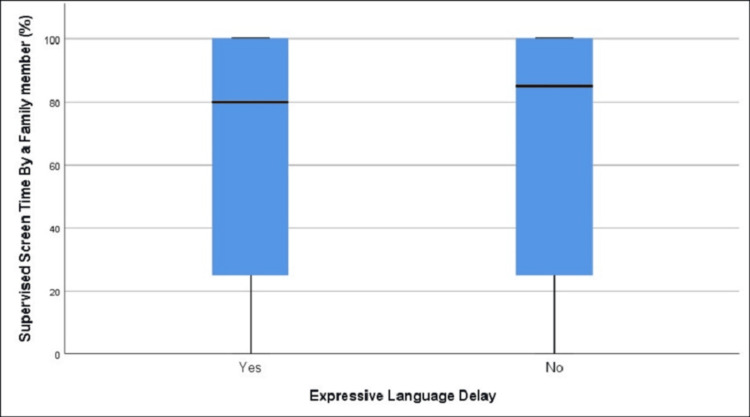
Distribution of supervised screen time in those with and without expressive language delay (age group 12-18 months)

The difference in mean supervised screen time percentage was not statistically significant between those with expressive language delay (63.19%) and those without it (63.25%), t = -0.02, df = 206.32, p = 0.985 (Table 2, Figure 2).

**Figure 3:**
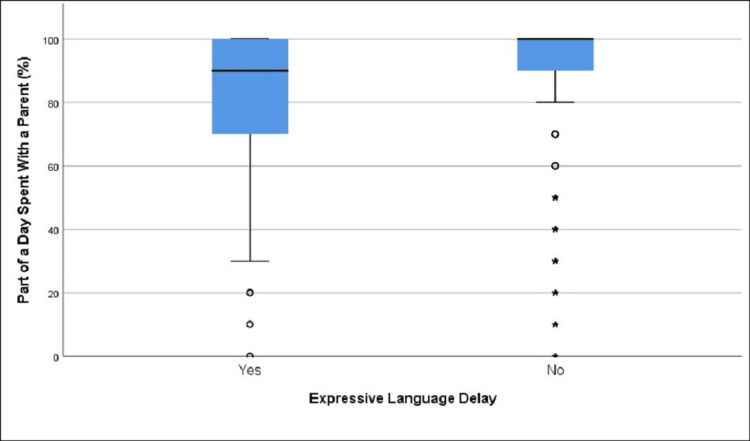
Distribution of part of the day spent with a parent in those with and without expressive language delay (age group 12-18 months)

We found that there was a statistically significant difference regarding the mean percentage of the day spent with a parent among children with (81.13%) and without (86.25%) expressive language delay, t = 2.60, df = 261.84, p = 0.01 (Table 2, Figure 3). 

19-30 months

**Figure 4:**
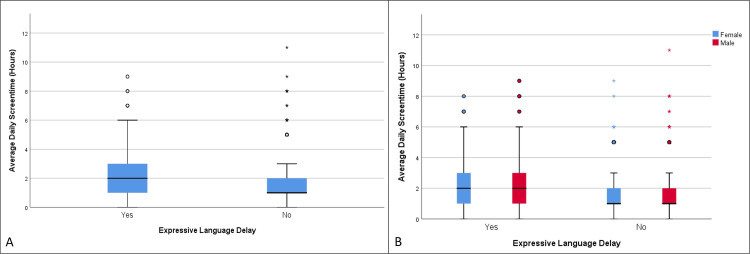
Distribution of average daily screen time in children with and without expressive language delay (A), as well as the same distribution categorized by biological sex (B) (age group 19-30 months)

There were statistically significant differences in the mean values of average daily screen time between children with expressive language delay (2.24 hours) and those without (1.58 hours), t = 7.47, df = 951.72, p < 0.001 (Table 4, Table 5, Figure 4). The OR for the development of expressive language delay among those exposed to screen time versus those not exposed was 1.79 (95% CI (1.32, 2.44)), p = 0.0002, and a statistically significant correlation was detected between exposure and the development of expressive language delay, χ²(1) = 14.30, p < 0.001. The OR was higher in males (OR = 1.85, 95% CI (1.27, 2.70)), p = 0.0013, compared to females (OR = 1.75, 95% CI (1.01, 3.03)), p = 0.04. In this age group, males (42.59%) were also more affected than females (26.66%), with a statistically significant difference (95% CI (11.03, 20.65)), χ²(1) = 39.17, p < 0.0001 (Table 6, Figure 4). However, average daily screen time was similar between boys and girls in this instance.

**Table 4:** Average daily screen time (ranging from 0, indicating no exposure, to 10 hours) and the number of children with expressive language delay (age group (age group 19-30 months)

		Expressive language delay		Total
		Yes	No	
Average daily screen time (hours)	0	64	185	249
	1	154	369	523
	2	124	219	343
	3	112	115	227
	5	51	32	83
	6	21	16	37
	7	5	2	7
	8	4	6	10
	9	2	1	3
	11	0	1	1
Total		537	946	1,483

**Table 5:** Overview of expressive language delay status, average daily screen time (in hours), percentage of supervised screen time, and percentage of time spent with a parent (age group 19-30 months)

	Expressive language delay	N	Mean	SD	Standard error of the mean
Average daily screen time (hours)	Yes	537	2.24	1.756	0.076
	No	946	1.58	1.452	0.047
Supervised screen time by a family member (%)	Yes	466	60.17	35.391	1.639
	No	749	64.36	35.525	1.298
Part of a day spent with a parent (%)	Yes	533	77.65	27.171	1.177
	No	943	79.94	25.742	0.838

**Table 6:** Average daily screen time, ranging from 0 (indicating no exposure) to 10 hours, and the corresponding number of males and females with expressive language delay (age group 19-30 months)

Biologic sex			Expressive language delay		Total
			Yes	No	
Female	Average daily screen time (hours)	0	18	80	98
		1	41	169	210
		2	36	100	136
		3	40	63	103
		5	14	20	34
		6	7	6	13
		7	3	0	3
		8	1	1	2
		9	0	1	1
	Total		160	440	600
Male	Average daily screen time (hours)	0	46	104	150
		1	112	199	311
		2	87	119	206
		3	72	52	124
		5	36	12	48
		6	14	10	24
		7	2	2	4
		8	3	5	8
		9	2	0	2
		11	0	1	1
	Total		374	504	878
Total	Average daily screen time (hours)	0	64	184	248
		1	153	368	521
		2	123	219	342
		3	112	115	227
		5	50	32	82
		6	21	16	37
		7	5	2	7
		8	4	6	10
		9	2	1	3
		11	0	1	1
	Total		534	944	1,478

**Figure 5:**
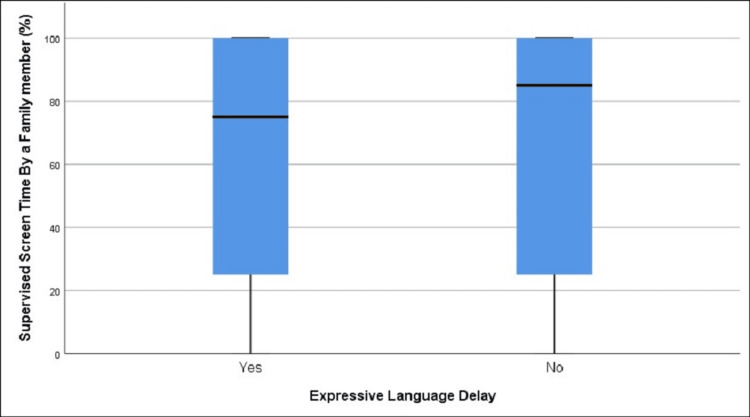
Distribution of supervised screen time in those with and without expressive language delay (age group 19-30 months)

Mean supervised screen time was higher in those who did not have expressive language delay (64.36% vs. 60.17%), and the difference was statistically significant, t = 2.00, df = 989.09, p = 0.046 (Table 5, Figure 5).

**Figure 6:**
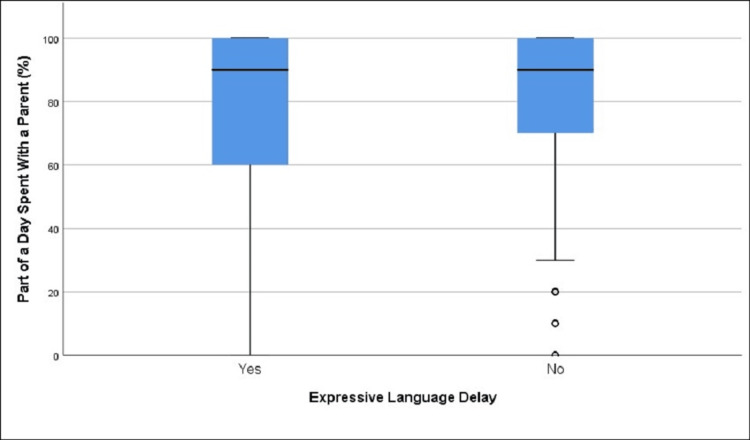
Distribution of part of the day spent with a parent in those with and without expressive language delay (age group 19-30 months)

In this age group, there was no statistically significant difference regarding the part of the day spent with a parent among children with (77.65%) and without (79.94%) expressive language delay, t = -1.57, df = 1055.29, p = 0.115 (Table 5, Figure 6).

31-48 months

**Figure 7:**
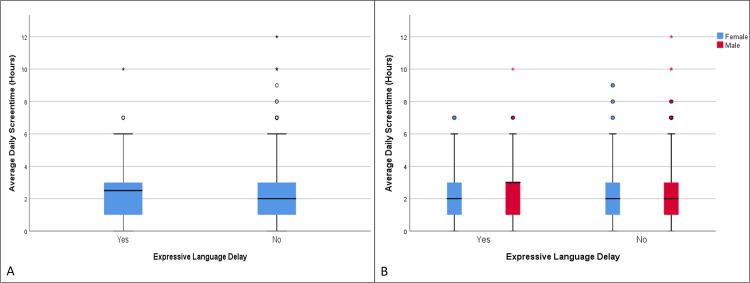
Distribution of average daily screen time in children with and without expressive language delay (A), as well as the same distribution categorized by biological sex (B) (age group 31-48 months)

Mean average daily screen time was statistically significantly different between children with expressive language delay (2.52 hours) and those without (2.12 hours), t = 2.53, df = 177.70, p = 0.012 (Table 7, Table 8, Figure 7). However, the OR for developing expressive language delay among those exposed to screen time versus those not exposed was 0.06 (95% CI (0.04, 0.09)), p < 0.0001, and a statistically significant association was found between no screen time exposure and expressive language delay, χ²(1) = 247.31, p < 0.001. This group also demonstrated that males (16.29%) were more affected than females (8.05%), with a statistically significant difference (95% CI (4.44, 11.89)), χ²(1) = 17.03, p < 0.0001 (Table 9, Figure 7). In this age group, average daily screen time exposure was not vastly different between boys and girls.

**Table 7:** Average daily screen time (ranging from 0, indicating no exposure, to 10 hours) and the number of children with expressive language delay (age group 31-48 months)

		Expressive language delay		Total
		Yes	No	
Average daily screen time (hours)	0	18	59	77
	1	28	354	382
	2	28	266	294
	3	50	226	276
	5	12	75	87
	6	8	14	22
	7	3	9	12
	8	0	2	2
	9	0	1	1
	10	1	2	3
	12	0	1	1
Total		148	1,009	1,157

**Table 8:** Overview of expressive language delay status, average daily screen time (in hours), percentage of supervised screen time, and percentage of time spent with a parent (age group 31-48 months)

	Expressive language delay	N	Mean	SD	Standard error of the mean
Average daily screen time (hours)	Yes	148	2.52	1.816	0.149
	No	1009	2.12	1.502	0.047
Supervised screen time by a family member (%)	Yes	147	47.28	35.78	2.951
	No	991	55.53	35.762	1.136
Part of a day spent with a parent (%)	Yes	148	73.24	28.433	2.337
	No	1010	72.31	26.811	0.844

**Table 9:** Average daily screen time, ranging from 0 (indicating no exposure) to 10 hours, and the corresponding number of males and females with expressive language delay (age group 31-48 months)

Biologic sex			Expressive language delay		Total
			Yes	No	
Female	Average daily screen time (hours)	0	6	26	32
		1	8	170	178
		2	7	117	124
		3	10	92	102
		5	4	31	35
		6	2	6	8
		7	2	1	3
		8	0	1	1
		9	0	1	1
	Total		39	445	484
Male	Average daily screen time (hours)	0	12	33	45
		1	20	183	203
		2	21	148	169
		3	40	132	172
		5	8	44	52
		6	6	8	14
		7	1	8	9
		8	0	1	1
		10	1	2	3
		12	0	1	1
	Total		109	560	669
Total	Average daily screen time (hours)	0	18	59	77
		1	28	353	381
		2	28	265	293
		3	50	224	274
		5	12	75	87
		6	8	14	22
		7	3	9	12
		8	0	2	2
		9	0	1	1
		10	1	2	3
		12	0	1	1
	Total		148	1,005	1,153

**Figure 8:**
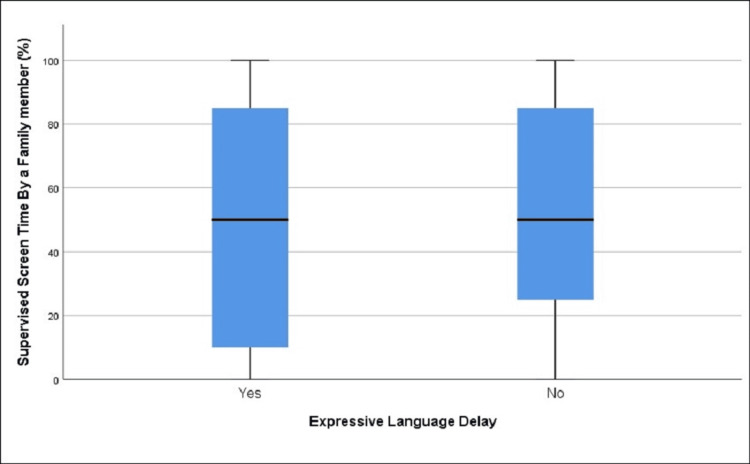
Distribution of supervised screen time in those with and without expressive language delay (age group 31-48 months)

We found a statistically significant difference in supervised screen time between children with (47.28%) and without (55.53%) expressive language delay, t = -2.61, df = 191.85, p = 0.01 (Table 8, Figure 8).

**Figure 9:**
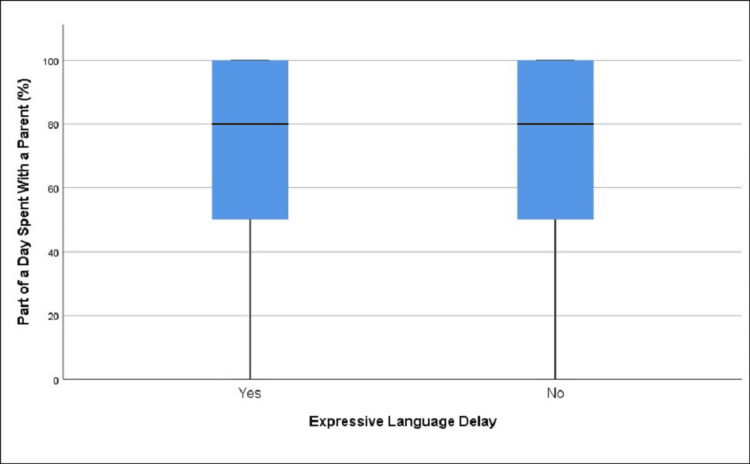
Distribution of part of the day spent with a parent in those with and without expressive language delay (age group 31-48 months)

In this age group, there was no statistically significant difference regarding the part of the day spent with a parent among children with (73.24%) and without (72.31%) expressive language delay, t = 0.394, df = 187.33, p = 0.707 (Table 8, Figure 9).

49-72 months

**Figure 10:**
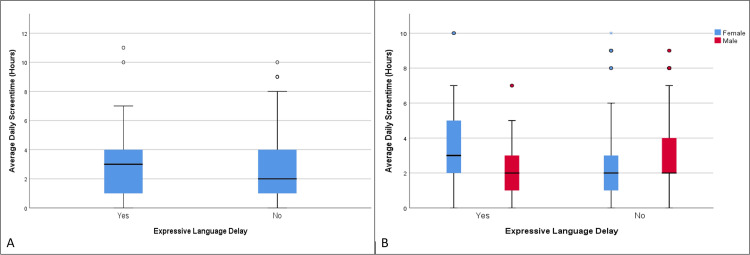
Distribution of average daily screen time in children with and without expressive language delay (A), as well as the same distribution categorized by biological sex (B) (age group 49-72 months)

There was no statistically significant difference in mean average daily screen time between children with expressive language delay (2.94 hours) and those without (2.59 hours) in this age group, t = 1.32, df = 77.54, p = 0.188 (Table 10, Table 11, Figure 10). The OR for developing expressive language delay among those exposed to screen time versus those not exposed was 0.51 (95% CI (0.13, 1.95)), p = 0.32, and the results were not statistically significant. No statistically significant association was detected between screen time exposure and language delay, χ²(1) = 1.00, p = 0.32. Additionally, there was no significant difference in the percentage of males and females affected (9.15% vs. 8.01%), with a 95% CI (-2.97, 5.03), χ²(1, N = 78) = 0.316, p = 0.5743 (Table 12, Figure 10).

**Table 10:** Average daily screen time (ranging from 0, indicating no exposure, to 10 hours) and the number of children with expressive language delay (age group 49-72 months)

		Expressive language delay		Total
		Yes	No	
Average daily screen time (hours)	0	3	16	19
	1	18	173	191
	2	14	220	234
	3	14	129	143
	4	7	100	107
	5	9	47	56
	6	1	19	20
	7	3	3	6
	8	0	7	7
	9	0	3	3
	10	1	1	2
	11	1	0	1
Total		71	718	789

**Table 11:** Overview of expressive language delay status, average daily screen time (in hours), percentage of supervised screen time, and percentage of time spent with a parent (age group 49-72 months)

	Expressive language delay	N	Mean	SD	Standard error of the mean
Average daily screen time (hours)	Yes	71	2.94	2.157	0.256
	No	718	2.59	1.574	0.059
Supervised screen time by a family member (%)	Yes	70	53.36	33.983	4.062
	No	708	52.97	33.595	1.263
Part of a day spent with a parent (%)	Yes	71	67.89	27.564	3.271
	No	717	65.51	25.987	0.971

**Table 12:** Average daily screen time, ranging from 0 (indicating no exposure) to 10 hours, and the corresponding number of males and females with expressive language delay (age group 49-72 months)

Biologic sex			Expressive language delay		Total
			Yes	No	
Female	Average daily screen time (hours)	0	1	3	4
		1	4	93	97
		2	5	100	105
		3	7	54	61
		4	2	35	37
		5	5	15	20
		6	1	6	7
		7	1	0	1
		8	0	1	1
		9	0	2	2
		10	1	1	2
	Total		27	310	337
Male	Average daily screen time (hours)	0	2	13	15
		1	14	80	94
		2	9	120	129
		3	7	75	82
		4	5	65	70
		5	3	32	35
		6	0	13	13
		7	1	3	4
		8	0	5	5
		9	0	1	1
	Total		41	407	448
Total	Average daily screen time (hours)	0	3	16	19
		1	18	173	191
		2	14	220	234
		3	14	129	143
		4	7	100	107
		5	8	47	55
		6	1	19	20
		7	2	3	5
		8	0	6	6
		9	0	3	3
		10	1	1	2
	Total		68	717	785

**Figure 11:**
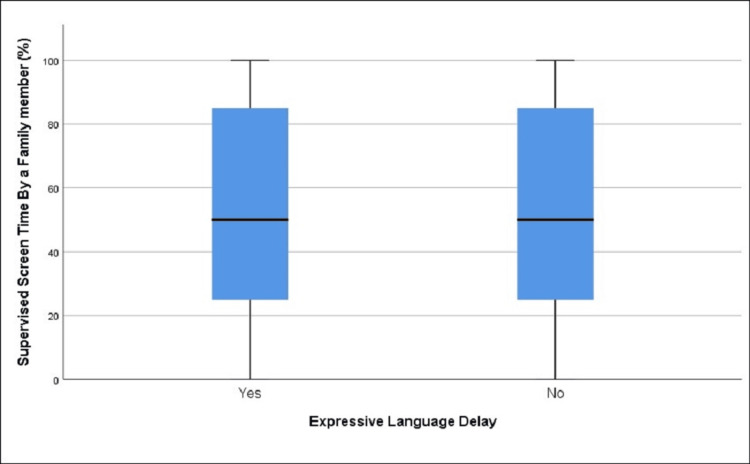
Distribution of supervised screen time in those with and without expressive language delay (age group 49-72 months)

No statistically significant difference in supervised screen time was detected among children with (53.36%) and without (52.97%) expressive language delay, t = 0.09, df = 82.90, p = 0.928 (Table 11, Figure 11).

**Figure 12:**
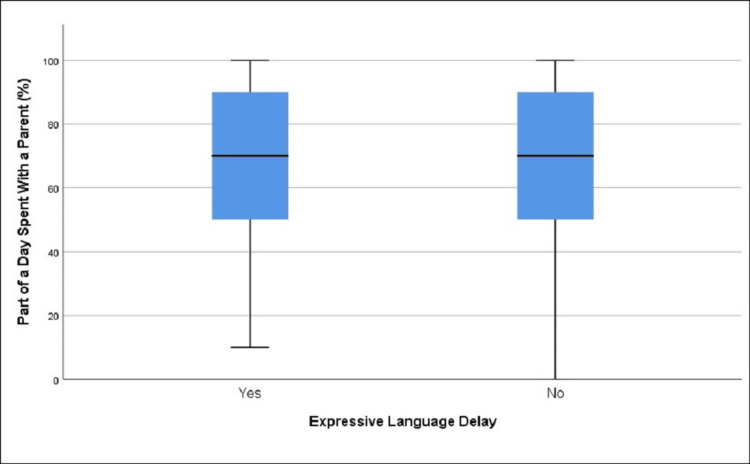
Distribution of part of the day spent with a parent in those with and without expressive language delay (age group 49-72 months)

No statistically significant difference was found in the part of the day spent with a parent, with children with expressive language delay at 67.89% and those without at 65.51%, t = 0.69, df = 82.80, p = 0.488 (Table 11, Figure 12).

## Discussion

The literature review highlights that the negative effects of screen time tend to dominate, despite evidence of both beneficial and detrimental impacts on child development. Numerous studies indicate that increased screen time can adversely affect a child’s language development. Although some research shows negligible or even positive effects, factors such as viewing duration, content quality, and parental co-viewing significantly influence outcomes [10]. One study underscores the importance of parental supervision and co-viewing in fostering language development, acknowledging that screen time presents both positive and negative influences on children’s language acquisition. Findings suggest that excessive early exposure to screens is harmful to language development, while later exposure can yield positive results [11].

A cross-sectional study involving children up to two years old in Bali, Indonesia, found that screen time exceeding two hours daily increased the risk of speech delay by 6.2 times, with male gender and low parental education as contributing factors [12]. Another article links increased screen time to mental health issues [13]. Additionally, a cohort study reported that greater screen time at one year of age correlated with developmental delays in communication and problem-solving skills at ages two to four [14]. Moreover, it was observed that approximately 31.1% of children with an average daily screen time of 3.1 hours experienced speech delays, suggesting that the later introduction of smart devices may lead to better language development outcomes [9]. A significant 90.3% of children with speech and language developmental delays were regular users of electronic devices [4].

Multiple studies indicate that being male is a potential risk factor for expressive language delay, as females typically develop these skills more rapidly [15,16]. However, it is essential to recognize that boys are often more vulnerable to conditions related to expressive language delay [17,18]. Consistently, research has demonstrated a higher prevalence and greater impact of expressive language delay among boys compared to girls [19]. This disparity may be partially attributed to greater screen time among boys, driven by access to video games, online tutoring, and educational quizzes [20].

While many studies highlight that gender is not the sole factor in speech delay, the quality and frequency of communication are crucial to the development of expressive language skills in males [21]. A study conducted in Iraq found no statistically significant difference in expressive language delay between male and female participants [22], a result that contrasts with existing findings [23]. This discrepancy raises questions about whether methodological issues or cultural factors may influence the results.

Several other risk factors for expressive language delay include low household income [24], low educational attainment among primary caregivers [25], family history of language acquisition issues [26], and postpartum depression (PPD) [27,28]. PPD is associated with various adverse effects on both parent and child, including an increased risk of expressive language delay due to reduced verbal interaction with infants who have yet to develop linguistic skills [27]. Similarly, low parental education levels contribute to expressive language delays, as parents with lower educational backgrounds typically use fewer words and simpler sentences [28].

Research suggests that low household income can indirectly impact expressive language development through stress, as families may prioritize basic needs over developmental aspects. Elevated stress can affect executive function and memory, potentially due to increased cortisol levels linked to changes in gray matter in areas such as the hippocampus, amygdala, and prefrontal cortex [29-36].

Some studies propose that interactive screen time may enhance learning, reinforcing the idea that not all screen time is detrimental and that interactive use, coupled with parental involvement, may be beneficial [37,38].

Limitations of this study include reliance on questionnaires completed by primary caregivers, which may affect data accuracy and interpretation. Additionally, the observational design restricts the ability to establish causal relationships between screen time exposure and language delay. It is important to note that the data were collected in the low-to-middle-income country of Georgia, where financial hardship is a recognized risk factor for expressive language delay.

The digitalization of daily activities seems unavoidable, especially in the wake of the COVID-19 pandemic. It is essential to assist parents and practitioners in navigating this transition to prevent unnecessary developmental delays, particularly expressive language delay, which is a significant concern for both caregivers and healthcare providers.

Data suggest a heightened risk of expressive language delay when children are exposed to screen time, particularly between the ages of 12-30 months. However, we cannot assert a significant difference in outcomes with increased average screen time. While screen time negatively affects both genders, data for the 12-18 months age group may indicate one of two possibilities: either girls show a higher tolerance for screen time compared to boys, or speech delays in boys are linked to other factors since girls were exposed to a statistically significantly different amount of average active daily screen time. Further investigation is necessary to identify the underlying factors contributing to this tolerance or to ascertain whether it relates to boys’ greater susceptibility to expressive language delay. Several longitudinal studies are currently being conducted globally to further explore the effects of early screen time exposure. Prior research has indicated a negative association between early screen time and later literacy, as well as reduced gray matter density in brain areas that influence language and cognitive skills [39,40].

In the 31-48 months age group, OR was 0.06, and chi-squared tests indicated a statistically significant correlation between the factors. Since the OR is less than 1, this suggests an inverse situation; in this age group, the absence of screen time exposure was linked to an increased risk of language delay. This finding aligns with evidence suggesting that a lack of screen time in older childhood may correlate with negative outcomes, such as social isolation and limited developmental opportunities [41].

While substantial evidence supports that parental supervision can mitigate the risks of screen time, our study did not find that higher levels of supervision consistently lead to such mitigation across all age groups. Nonetheless, research indicates that active supervision and engagement during screen time can enhance language and cognitive outcomes [42].

Our data indicate that most parents do not adhere to established screen time recommendations. Exposure to screen time in children aged 12-30 months is linked to an increased risk of expressive language delay for both genders. The higher prevalence observed in boys may be attributable to screen time exposure, other factors, or a combination thereof; however, this remains uncertain and warrants further exploration. Additionally, we found that a lack of screen time exposure may be associated with an increased risk of language delay in children aged 31-48 months.

## Conclusions

This study provides significant insights into the complex relationship between screen time exposure and expressive language development in young children. Our findings reveal that early screen time exposure is associated with a higher risk of expressive language delay. The data suggest that children aged 12-30 months are especially vulnerable. However, we cannot definitively state that the amount of average daily screen time is significantly changing the outcome. Conversely, we also found that a lack of screen time exposure may be associated with an increased risk of language delay in children aged 31-48 months, highlighting the nuanced role of screen time in child development. Parental supervision is a critical factor; however, our study indicates that higher levels of supervision do not consistently mitigate the risks associated with screen time across all age groups. Further research is warranted to explore the underlying factors contributing to the observed differences in susceptibility to expressive language delay among genders and to examine the long-term impacts of early screen exposure. By understanding these dynamics, we can better support children’s developmental needs in an increasingly screen-saturated environment.

## Appendices

**Table 13:** Survey samples used throughout the research

Survey – 12-18 months		
Question	Answers	
Write the initials of the child (for example K.J.)		
Date of Birth (Day/Month/Year)		
Select the sex of the child	Female	Male
Age of the child (in months):		
What is the average active daily screen time exposure in hours? (include every device, 0 means no screen time exposure)		
What percentage of active screen time is supervised? (0 means no supervision)		
What part of the day is spent with a primary caregiver? (in percentages) (0 means no time spent)		
Does child call for a parent? (Mama, dada etc.)	Yes	No
Does child use dada, mama or other variation to request something?	Yes	No
Does child use gestural communication?	Yes	No
What is the vocabulary of the child besides mama and dada? count the words that child has invented as long as they are used to describe something		
Survey – 19-30 months		
Question	Answers	
Write the initials of the child (for example K.J.)		
Date of Birth (Day/Month/Year)		
Select the sex of the child	Female	Male
Age of the child (in months):		
What is average active daily screen time exposure in hours? (include every device, 0 means no screen time exposure)		
What percentage of active screen time is supervised? (0 means no supervision)		
What part of the day is spent with a primary caregiver? (in percentages) (0 means no time spent)		
Does child use at least 5 words to request something or name objects?	Yes	No
Does child use two-word combinations?	Yes	No
Does child use any other gesture than pointing fingers or waving hand?	Yes	No
Does child play in close proximity and/or with other children?	Yes	No
What is the vocabulary of the child besides mama and dada? count the words that child has invented as long as they are used to describe something		
What part of the child's vocabulary is intelligible by strangers? (in percentages)		
Survey – 31-48 months		
Question	Answers	
Write the initials of the child (for example K.J.)		
Date of Birth (Day/Month/Year)		
Select the sex of the child	Female	Male
Age of the child (in months):		
What is average active daily screen time exposure in hours? (include every device, 0 means no screen time exposure)		
What percentage of active screen time is supervised? (0 means no supervision)		
What part of the day is spent with a primary caregiver? (in percentages) (0 means no time spent)		
Does child engage in dialogue? (at least two exchanges)	Yes	No
Does child ask questions? (for example: where is father? where is mother?)?	Yes	No
Does child say their name?	Yes	No
Does child play with other children?	Yes	No
Can child describe action which is happening in an image? (running, eating, driving or etc.)	Yes	No
Can child say sentence with 3 or more words?	Yes	No
Can child learn new words from listening to music, a story, or a movie?	Yes	No
What is the vocabulary of the child besides mama and dada? count the words that child has invented as long as they are used to describe something		
What part of the child's vocabulary is intelligible by strangers? (in percentages)		
Survey – 48-72 months		
Question	Answers	
Write the initials of the child (for example K.J.)		
Date of Birth (Day/Month/Year)		
Select the sex of the child	Female	Male
Age of the child (in months):		
What is average active daily screen time exposure in hours? (include every device, 0 means no screen time exposure)		
What percentage of active screen time is supervised? (0 means no supervision)		
What part of the day is spent with a primary caregiver? (in percentages) (0 means no time spent)		
Does child engage in dialogue? (at least three exchanges)	Yes	No
Can child tell at least 2 stories which he/she came up with or heard from someone else? (for example: a cat was saved by a firefighter)	Yes	No
Does child say their name?	Yes	No
Can child answer 2 or 3 questions about a story that you read or told to them?	Yes	No
Can child observe the rules of playing while playing with other children?	Yes	No
Can child say sentence with 4 or more words?	Yes	No
Can child learn new words from listening to music, a story, or a movie?	Yes	No
What is the vocabulary of the child besides mama and dada? count the words that child has invented as long as they are used to describe something		
What part of the child's vocabulary is intelligible by strangers? (in percentages)		
